# Supplementary material for: Effect of Danofloxacin Treatment on the Development of Fluoroquinolone Resistance in Campylobacter jejuni in Calves
Source: Antibiotics (Basel). 2022 Apr 15;11(4):531. doi: 10.3390/antibiotics11040531 (PMC9025843; doi:10.3390/antibiotics11040531)
Supplement: Supplementary file 1 [file antibiotics-11-00531-s001.zip › antibiotics-1654434-supplementary.pdf]

antibiotics

Supplementary Materials

Table S1. Bacterial isolates used for inoculation of calves with *Campylobacter jejuni* (oral) and *Mannheimia haemolytica* (trans-tracheal) in the current study.

Isolate/strain	Source	Origin	Isolation date	Reference
C. jejuni IA-6-FC-30	Feces of healthy cattle	Iowa	2013	[67]
C. jejuni MO-2-FC-25	Feces of healthy cattle	Missouri	2013	[67]
M. haemolytica VDL4004	Pneumonic lung of calf	Iowa	2018	This study
